# Letermovir for the compassionate therapeutic use of cytomegalovirus infection

**DOI:** 10.1007/s10096-020-03990-w

**Published:** 2020-09-11

**Authors:** Lorenz Schubert, Lisa Fisecker, Florian Thalhammer, Heinz Burgmann, Christoph Steininger

**Affiliations:** Division of Infectious Diseases and Tropical Medicine, Department of Medicine I, Medical University Vienna, Vienna, Austria

**Keywords:** Cytomegalovirus infection, Letermovir, Compassionate therapeutic use, Viral kinetics

## Abstract

**Purpose:**

Data on the efficacy, dosing and safety of letermovir for the compassionate therapeutic use of CMV infections are limited.

**Methods:**

Clinical and virological efficacy of letermovir was assessed in a retrospective single-centre study of patients who received letermovir for the compassionate therapeutic use of CMV infections.

**Results:**

Letermovir initiation yielded prompt treatment response in 7 out of 9 patients (77.7%).

**Conclusion:**

Letermovir may be an effective and well tolerated option in the compassionate treatment of CMV infections, although recurrence of CMV and emergence of resistance may be issues.

## Background

Cytomegalovirus (CMV) infections remain a prevalent cause for morbidity and mortality in immunosuppressed solid-organ and bone-marrow transplant patients [1]. The first line therapeutic agents, ganciclovir (GCV) and its oral prodrug valganciclovir (VGCV), inhibit CMV replication by targeting the viral DNA polymerase pUL54. However, haematological side effects limit its therapeutic potential in 10–20% of the cases. Furthermore, genetic mutations of *UL97* and *UL54*, conferring antiviral resistance, were reported, especially in cases of prolonged antiviral therapy, lack of prior CMV immunity in transplant patients and strong immunosuppression [2].

In cases of clinical or virological treatment failure, guidelines recommend the escalation of valganciclovir dose or escalating to cidofovir or foscarnet [1]. Both options are often limited by the toxic profile. Furthermore, cross-resistances were reported [2]. Hence, novel drugs that target alternative viral mechanisms are urgently required. Recently, letermovir, a new antiviral compound, was approved for the prophylaxis of CMV disease in bone-marrow transplant recipients [3]. Letermovir inhibits the terminase complex, which is essential for viral replication, for cleavage and packaging of large concatemers of CMV-DNA [4].

According to its licence, letermovir was mainly used as a prophylaxis agent for CMV infections. However, its different mechanism of action as well as its excellent side effect profile made it an appealing off-label option for the therapy of GCV resistant CMV infections, as well was for patients who reported severe leukopenia or reduced kidney function. However, data are limited to case series [5–7].

In the present report we document a case series of patients who have received letermovir for the compassionate use for CMV infections, highlighting its antiviral potential, but also pointing out possible difficulties, thus judging its efficacy in the clinical setting.

## Materials and methods

### Subjects and data collection

The present retrospective single-centre study exclusively comprises patients suffering from CMV infections who received letermovir for the compassionate use for treatment of CMV infections. The study protocol was approved by the Ethics Committee of the Medical University of Vienna, Austria (ECS 2013/2019), and all study-related procedures were conducted according to the declaration of Helsinki. We included adult patients who got diagnosed with CMV infection, defined as CMV DNA copy levels > 200 IU/ml measured in plasma and were treated with letermovir due to ganciclovir refractory or resistant CMV viremia or patients who were intolerant to receive ganciclovir or foscarnet. Intolerance to receive ganciclovir or foscarnet was decided by the responsible team of clinicians.

The same dosage of LMV was used for treatment as for prophylaxis of CMV infection—480 mg qd or 240 mg if on concomitant cyclosporin [3]. As suggested in the international consensus guidelines, CMV infection was separated into three groups: asymptomatic infections, viral syndrome, or tissue invasive (“end organ”) disease [1]. CMV syndrome and tissue invasive disease were diagnosed according to Ljungman et al. [8]. Treatment was monitored closely, and patients were regularly controlled for adverse events or development of viral syndrome or tissue invasion. Baseline demographics, CMV associated symptoms, previously received CMV prophylaxis or therapy, viral kinetics, ongoing immunosuppression and clinical outcome were retrospectively collected. Clinical response was defined as a decline of viral load to < 200 IU/ml. Furthermore, viral half-life was calculated, according to the formula t1/2 = −ln2/slope [9]. Patients who achieved the endpoint were further controlled for reactivation of CMV.

## Results

A total of 11 patients were identified who received letermovir for the compassionate use for CMV infection. One patient had to be excluded as the exact time of termination of treatment was not documented. Another patient was excluded as she was already discussed in an earlier case report, which has not yet been published. The other nine patients are discussed below. Table 1 gives an overview of the demographic characteristics. The median age was 66 years (45–70), and 77.8% of the patients was male. Five patients were solid organ transplant recipients (55.6%), two developed CMV infection after HSCT (22.2%), one patient suffered from TARFO syndrome (11.1%) and one patient suffered from systemic lupus erythematosus (SLE) (11.1%). Six patients experienced asymptomatic CMV viremia (66.7%), one CMV syndrome (11%), one a probable CMV pneumonia (11%) and one patient with probable CMV enteritis (11%) [8]. Clinical reasoning for compassionate use of letermovir was as follows: confirmed antiviral resistance against GCV (*n* = 2, 22.2%), virological treatment failure of GCV (*n* = 1, 11.1%) and HSCT associated with significant CMV viremia (*n* = 2, 22.2%). In the other four patients, the clinicians opted for letermovir instead of ganciclovir or foscarnet, to prevent aggravation of coexisting diseases. In three of the patients severe leukopenia and concomitant infection (two of them septic) were the reasons for letermovir selection. Finally, one patient (11.1%) suffering from a multicentric form of Castleman disease called TAFRO syndrome (an acronym for thrombocytopenia, anasarca, myelofibrosis, renal dysfunction and organomegaly), experienced CMV reactivation under therapy with high-dose cortisone and tocilizumab. To prevent further aggravation thrombocytopenia, which would have ultimately led to discontinuation of tocilizumab, clinicians decided for compassionate use of letermovir instead of valganciclovir.

**Table 1:** Characteristics of the patients treated with letermovir

Patients	Demographics	Immuno-suppression	Presentation of CMV	Previous antivirals	Indication for LMV	Daily dose of LMV (mg)	Adaption of immuno-suppression	Initial CMV DNA IU/ml	Time to suppression of virus replication (days)	Recurrence of CMV infection
Patient 1	f, 69a, kidney transplantation	MMF, TAC and cortisone	Asymptomatic	VGCV	Leukopenia and GCV refractory viremia	480	MMF stopped	833	8	No
Patient 2	m, 60a, HSCT	MMF, CsA and cortisone	Asymptomatic	None	Prevent aggravation of leuko- and thrombocytopenia after HSCT	240	MMF paused	261	14	Yes
Patient 3	m, 70a, heart transplantation	MMF, TAC and cortisone	Probable pneumonia	GCV^1^	Leukopenia under GCV and sepsis	480	MMF paused	898	Not achieved	
Patient 4	m, 58a, heart transplantation	MMF, TAC and cortisone	Asymptomatic	VGCV	Confirmed resistance	480	None	39,600	185^2^	No
Patient 5	m, 74a, kidney transplantation	MMF and TAC	CMV syndrome	VGCV	Confirmed resistance	480	MMF paused	2770	34	Bo
Patient 6	m, 45a, TARFO-syndrome	tocilizumab and cortisone	Asymptomatic	None	Prevent aggravation of thrombocytopenia under tocilizumab	480	None	1610	23	No
Patient 7	m, 68a, kidney transplantation	MMF, TAC and cortisone	Asymptomatic	None	Leukopenia and sepsis	480	MMF dose reduction	1550	29	No
Patient 8	f, 65a, SLE	MMF and cortisone	Probable enteritis	None	Prevent aggravation of leukopenia	480	None	16,000	83	No
Patient 9	m, 61a, HSCT	MMF and CsA	Asymptomatic	None	Prevent aggravation of leuko- and thrombocytopenia after HSCT	240	None	224	23	No

*f* female, *m* male, *a* age, *HSCT* haematopoietic stem cell transplantation, *SLE* systemic lupus erythematosus, *MMF* mycophenolate mofetil, *TAC* tacrolimus, *CsA* cyclosporine A, *LMV* letermovir, *VGCV* valganciclovir, *GCV* ganciclovir

^1^CMV IgG co-administration

^2^In patient 4 letermovir treatment was prematurely discontinued at CMV DNA load 739 IU/ml, and later on re-administered. Although treatment course was prolonged (185 days) no signs of CMV end-organ disease were reported

**Fig. 1:**
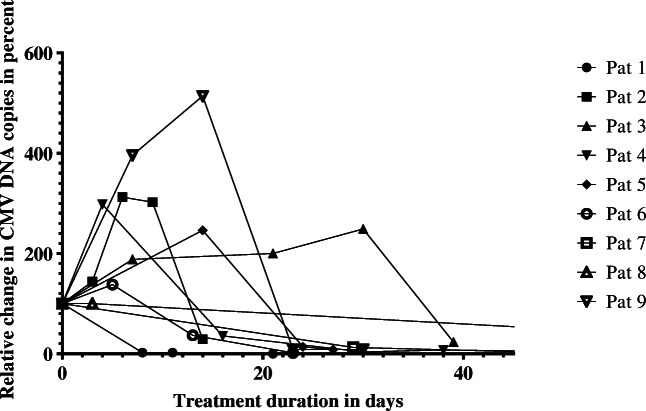
Relative change of CMV DNA copies in percent after initiation of letermovir treatment. The average increase was 2.7-fold (SD = 1.3). Final descent, defined by the definite negative movement, was seen as late as day 30 (mean = 8.7, SD = 8.8)

Initiation of letermovir treatment yielded a viral response, after an initial viral load increase, in 7 out of 9 patients (77.8%). The median duration of letermovir treatment was 31 (8–127) days. The median duration to achieve a decrease of viral load < 200 IU/ml was 23 (8–83) days. Viral kinetic curves are shown in Fig. 1. In the other two patients (patients 3 and 4), treatment was discontinued prematurely at CMV DNA copy levels > 200 IU/ml, at 211 IU/ml and 739 IU/ml respectively. Both patients experienced an increase of viral load within the next month, and letermovir was re-administered. After re-administration, patient 4 experienced a slow but steady decrease of viral load, resulting in CMV DNA copies < 200 IU/ml within a total of 185 days of treatment. Re-administration of letermovir in patient 3 yielded a decrease of CMV copy load, but general estate of the patient further aggravated and the patient developed sepsis. In a discussion with the family treatment discontinuation was decided and the patient died within the following days. End-organ disease occurred in none of the asymptomatic CMV patients under letermovir treatment, but one patient (patient 2) experienced a CMV end-organ disease only 2 weeks after treatment discontinuation. The episode was subsequently treated with VGCV, as leukopenia had improved.

The initiation of letermovir therapy was associated with an initial increase of viral load in 7 out of 9 (77.8%) patients. The average increase was 2.7-fold (SD = 1.3) of the viral load at start of treatment. Final descent, defined by the definite negative movement, was seen as late as day 30 (median = 6, range [3–30]). The viral half-life was 7.1 (1.49–9.1) days.

## Discussion

The present report demonstrates the potential of letermovir for the effective treatment of CMV infections. All present patients received letermovir as a monotherapy. The therapy was generally well tolerated, and no adverse events were reported. Treatment initiation yielded a decrease of viral load to < 200 copies/ml in 88.9% of the patients. However, one patient with complete clinical response experienced CMV end-organ disease within 2 weeks after treatment discontinuation. Immunosuppression was adapted in all SOT patients, by either dose reduction or pausing of cell-cycle inhibitor.

Therapeutic strategies for clinical or virological failure of CMV infections include the escalation of valganciclovir dose or switching to cidofovir or foscarnet [1]. However, both drugs are associated with the potential for severe adverse events, such as nephrotoxicity and myelosuppression, which limit their therapeutic usefulness. Letermovir, a novel inhibitor of the terminase complex, was recently approved for the prophylaxis of CMV infection in allogenic HSCT, but is not yet approved for pre-emptive therapy [3]. Its favourable side effect profile has led to an increased off-label use for GCV-resistant CMV infections or if the patients were intolerant to receive other treatment options. However, first observations of letermovir resistance have been reported [10–13]. A recently published letermovir resistance analysis among HSCT recipients receiving letermovir prophylaxis identified all letermovir resistance associated variants within the UL 56 gene [13]. Although some treatment durations were prolonged, no viremia breakthrough was reported in our cohort.

Dosing of letermovir for preemptive therapy remains uncertain, as there is no approval for letermovir in this indication. Stoelben et al. successfully used lower letermovir doses of 40 bid or 80 qd for preemptive therapy in kidney transplant recipients, whereas Turner et al. used higher doses of up to 960 mg qd for the salvage therapy of drug-resistant CMV retinitis without an emergence of adverse events [6, 14]. Letermovir dose in our cohort coincided with the dose recommendations for the prophylaxis of CMV infection, 480 mg qd or 240 mg if on concomitant cyclosporin [3]. The same treatment protocol was recently chosen by Phoompoung et al. for the salvage therapy of CMV infections in transplant recipients [7].

The average duration until decrease of viral load to under < 200 mg/ml was 32.9 days. The viral half-life time under letermovir was 6.3 days, which is longer than previously published viral half-lives of solid-organ transplant patients treated with GCV [9]. As demonstrated in earlier reports, the viral load initially increases after therapy start. The average increase in our cohort was 2.7-fold of the viral load at treatment start. However, the increase in viral load was not associated with an increase in symptoms. This may be explicable by the mode of action of letermovir, as viral replication is blocked at a late stage, possibly yielding high intracellular titre of viral DNA in absence of a viable virus. This emphasizes the need for alternative methods to estimate the risk of CMV disease under letermovir treatment.

The present study has some limitations. As a result of the retrospective study design, the included cohort is heterogenous, leading to a broad spectrum of indication for letermovir. However, precisely this highlights the clinicians’ desire for alternative treatment options, as existing first-line therapies are often limited by their severe side effect profile, especially in patients with concomitant bacterial infections. Further, we could not exclude that the patient’s immune status contributed significantly to the treatment outcome, especially in patients with long lasting viremia.

Hence, prospective trials evaluating efficacy, safety, drug dosing, treatment duration and emergence of drug resistance are urgently required.

## Data Availability

The datasets generated during and/or analysed during the current study are available from the corresponding author on reasonable request.
